# Development of an upper limb passive exosuit for the 2023 ASTM Exo Games

**DOI:** 10.3389/frobt.2024.1485177

**Published:** 2024-11-27

**Authors:** Stijn Kindt, Elias Thiery, Stijn Hamelryckx, Adrien Deraes, Tom Verstraten

**Affiliations:** ^1^ Brubotics, Vrije Universiteit Brussel, Brussels, Belgium; ^2^ Department of Mechanical Engineering (MECH), Vrije Universiteit Brussel, Brussels, Belgium; ^3^ Robotics and Multibody Mechanics Research Group, Flanders Make, Brussels, Belgium

**Keywords:** occupational exoskeletons, industrial exoskeletons, exosuit, exoskeleton, Exo Games, standards

## Abstract

This paper presents the design of the passive upper limb exosuit that won the design competition in the 2023 ASTM Exo Games. The tasks were first analyzed to provide information about the requirements of the design. Then a design was proposed based on the HeroWear Apex exosuit but with improvements from the competition team members. The four tasks of the competition are discussed in detail, including good and poor execution practice. Experiments are performed to measure the forces generated in the elastic elements that support the back and the ones that support the arms. Flex tests are also discussed to show that the exosuit does not hinder the movement of the user in a meaningful way when it is switched off. The performance during the tasks is discussed and based on this and designs of competitors, improvements to the overall design are proposed for future versions.

## 1 Introduction

Exoskeletons are wearable devices used to carry some of the load from physical activities. Exoskeletons have three use cases: rehabilitation, where it helps the patient to regain their muscle strength, assistive exoskeletons, which aid physically impaired persons with daily activities (Kim et al., 2017) and occupational exoskeletons which are used to support some of the load. Several studies have already investigated the possible health benefits of using an occupational exoskeleton in e.g. farming (Upasani et al., 2019), palletization (Abdelmomen et al., 2019), construction jobs (De Vries et al., 2022) and factory work (Aida et al., 2009).

An exosuit is a special type of exoskeleton that has no rigid kinematic components (Babič et al., 2021). Exosuits have better user acceptance in occupational exoskeletons.

Different kinds of exoskeletons and exosuits exist on the market that provide a varied range of support locations and actuator types (Zhu et al., 2021). The most prominent types of support are back, arm/shoulder and leg exosuits. Both exoskeletons and exosuits are commercially available for occupational use (Koopman et al., 2019; Goršič et al., 2021).

Exoskeletons and exosuits can be classified as being active, passive or semi-active (Toxiri et al., 2019; Crea et al., 2021), where passive elements are coupled or decoupled or their mechanical properties (e.g. stiffness of a beam spring) are changed automatically during use. Active means that there is some kind of actuation that provides power to the body from an external source of power. Different kinds of actuation are possible, (Kim et al., 2017; Aida et al., 2009; Luo and Yu, 2013), but since all of these require an external power source, the available time to work with the device will be limited and recharges will be required. As an alternative, some active devices are attached to an external power outlet or pneumatic system. This limits the working area of the device due to cabling and such. Passive exosuits require no external power source and can be used for as long as the user wants which is an advantage over active systems.

Passive exoskeletons use only the power provided by the body. This is done by storing the energy generated by the motion of the body in one part of a movement and releasing it when it is needed more. Passive exoskeletons often use springs to store this energy. Both stiff spring and soft spring (Toxiri et al., 2019) examples exist. In the case of the soft springs elastic bands are the most common way of storing the potential energy (Abdoli et al., 2006). For the stiff springs gas springs (Koopman et al., 2019) and coil springs are common use cases. The soft springs have the benefit of more compliance towards the user and will in general be more comfortable. The stiff springs can provide more support, but will be more limiting to the freedom of motion of the user. This issue can be somewhat mitigated by including a mechanism that allows the springs to be disengaged.

Passive exosuits are mainly used for lifting and static holding tasks in industry (De Looze et al., 2016). These exosuits are usually upper body devices that focus on the back and shoulder of users. Examples of tasks are the lifting of loads in warehouses where back support is usually applied. The attempt here is to avoid spinal compression and prevent long and short term back injuries. Overhead tasks like drilling or parts assembly in automotive industry is where shoulder support can shine since it prevents fatigue in users.

Standardization of exoskeletons and exosuits has been attempted before (Li-Baboud et al., 2019; Pesenti et al., 2021; Pinto-Fernandez et al., 2020) but a standardized way to test these devices is still not widely accepted (Pinto-Fernandez et al., 2020), although efforts are still ongoing. The human factor that is always present when testing exoskeletons and exosuits is one of the reasons why finding a widely accepted standardized way of testing is difficult for exosuits and exoskeletons. ASTM is one of many organisations that develop standards for a variety of different fields and applications. They took on the challenge of finding a standardized way of testing exoskeletons and exosuits in committee F48 (ASTM, 2024). To test if the standards that were developed in this committee make sense, the Exo Games are set up to allow teams of student to compete against each other in tasks that are derived from the ASTM developed standards. This also motivates students to enter the field of exoskeletons, which is an upcoming field in industry as well (Mohamed Refai et al., 2024).

This paper presents the design that won the design award of the 2023 ASTM Exo Games. It explains the choices that were made during the design process and analyzes the performance of the exosuit during the four different tasks. The latter is supported by a number of experiments representative of common movements. Some improvements based on what was learned during this performance and on the designs of the competitors are then proposed and discussed.

The paper is structured as follows. In Section 2, we present the tasks that had to be performed during the competition. In Section 3, the design requirements are investigated and the design of the exosuit based on these requirements is discussed. Sections 4, 5 discuss the experiments that were performed on the exosuit. In Section 5, the performance of the exosuit during the competition is explained. Section 6 discusses the design based on the performance during the tasks and on the designs of the competitors. Section 7, concludes the paper with a take home message about the design discussed in previous sections.

## 2 The Exo Games competition

### 2.1 The tasks

**FIGURE 1:**
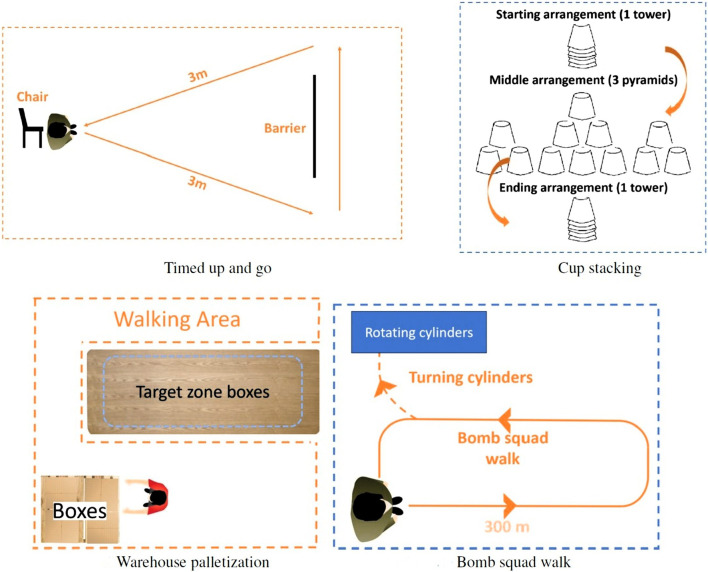
Graphical explanation of the four different tasks that have to be performed during the competition.

**FIGURE 2:**
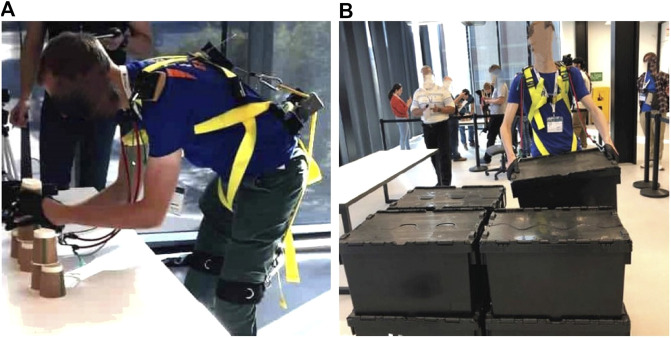
Images of some of the tasks being performed during the actual competition. **(A)** Cup stacking. Small paper cups are used in the competition. These cups are difficult to seperate. **(B)** Warehouse palletization. Boxes are stacked in two layers on the pallet.

There were four tasks in the competition that the user wearing the exoskeleton had to perform. Each of these tasks are designed to test the exoskeleton in different ways. The tasks are derived from ASTM standards on exoskeletons and exosuits (ASTM, 2024). Examples of good and poor execution are given for each task, which are based on the competition guidelines and remarks made by the judges. Figures 1, 2 concisely illustrate each task.

#### 2.1.1 Timed up and go

The first task is the timed up and go. The task consists of starting from a sitting position in a chair and standing up. The test subject walks 3 m and will sit down in the same chair again. The user should go from sit to stand and *vice versa* without any movement of the chair. To evaluate this the chair is placed in a square. As long as the chair does not move outside of the square it is considered as no movement. The main goal of this test is to see if the exoskeleton will hinder the test person in any way when performing tasks that are easy for the average person without an exoskeleton.

##### 2.1.1.1 Good execution

The chair does not move when the test subject gets up. The user maintains a good posture when standing up with a straight back and without twisting his body. The user then walks forward until he has walked the required length of 3 m. The user places special attention on his speed and makes sure that he never runs during the task. The user then walks back to the chair and sits down without causing any kind of movement of the chair. A good ergonomic posture is maintained during walking and sitting back down.

##### 2.1.1.2 Poor execution

The user gets out of the chair and causes so much movement of the chair that it moves outside the taped off square surrounding the chair. The user runs when covering the required distance and moves the chair a second time when sitting down again. During the task the user maintains a bent back and when getting out of the chair he needlessly twists his body.

#### 2.1.2 Warehouse palletization

A second task is the warehouse palletization test. It simulates a worker in a warehouse that has to lift boxes weighing a maximum of 18 kg. The boxes have to be stacked from a pallet onto a table with a height of 76 cm. Boxes need to be carried at torso level during transport. More than one box can be carried at a single time as long as this can be done safely. The boxes are carried over a distance of 305 cm before they can be placed down. After all of the boxes have been placed on the table they need to be carried back to the pallet in the same manner as before. During this task, special attention goes to the posture of the person.

##### 2.1.2.1 Good execution

The test subject bends through his knees and maintains a good ergonomic posture when lifting the heavy loads. One box is carried at a time to the table at a good pace without running. When putting down the load the user does not twist his body or bends over the table to get extra reach. Instead the user walks around the table if needed so a good posture can be maintained. When picking up the boxes from the table the user slides the boxes towards him so there is no need to reach forward to grasp the handles. The boxes are placed on the pallet again using a squatting technique to keep good posture.

##### 2.1.2.2 Poor execution

The user picks up the boxes by bending forwards putting an unnecessarily large load on the spine. The user then runs with the boxes towards the table and places them on the table. The test subject reaches across the table to put the boxes further on it. The same techniques are used when the boxes are transported from the table back towards the pallet.

#### 2.1.3 Cup stacking

A third test consists of cup stacking. Here the test person needs to use paper cups in order to create three separate pyramids. The first one consisting of three cups, then six cups and finally three cups again. The pyramids have to be torn down afterwards. The task should be completed as quickly as possible and is present in the competition to see if the exosuit is hindering the user or is uncomfortable in any way.

##### 2.1.3.1 Good execution

The user maintains a straight back and good posture during the cup stacking and resists any temptations to lean forward. The cup stacking task is performed quickly and fluently without any interference from the exoskeleton.

##### 2.1.3.2 Poor execution

The user leans forward across the cups and performs the task with a bad posture. During the task the exoskeleton hinders the user and limits his movement.

#### 2.1.4 Bomb squad walk

The final task is the bomb squad walk. Here the test person needs to hold a weight of 7 kg in each hand. A vest weighing 11 kg has to be worn either under or over the exoskeleton. The person has to walk 300 m with these loads until he reaches a task to test the agility. This task consists of unscrewing pipes from a low wooden contraption on the floor. The wooden contraption cannot be touched by the person or exoskeleton. This tests the agility of the user in the exoskeleton. Finally the same distance of 300 m has to be walked again. The time in which this task is completed is only a factor of the evaluation. The posture during carrying is equally important. A good exoskeleton should not force the body of a user in uncomfortable and bad ergonomic positions.

##### 2.1.4.1 Good execution

The user maintains a straight back and his arms close to his body while walking with the weights. A good walking pace is maintained during the task. When the wooden contraption is reached the test subject attempts to move around it to reach all of the pipes that need to be turned and tries to bend through his knees as much as possible. The user then walks again keeping the same good posture until the finish line is reached.

##### 2.1.4.2 Poor execution

The user walks with a bent back and his arms far from his body requiring additional effort and forcing the body into a bad posture that loads the spine unnecessarily. The user runs with the weights until reaching the wooden contraption. Here the user bends forward to reach all of the pipes, sometimes needing to touch the contraption. The user then runs to the finish line in the same bad posture as before.

### 2.2 Competition requirements

When designing the exoskeleton, certain guidelines set by ASTM were to be followed.

#### 2.2.1 Interface

The exoskeleton must have as basis for attaching to the body a certified safety harness. Other attachments can be added as well if so desired. This is to ensure the exoskeleton is safe, but also to create a sort of equal starting point for all teams. In the specifications for the 2024 edition of the Exo Games, this requirement was dropped.

#### 2.2.2 Cost

Another requirement imposed by the competition rules was that the total cost of all materials for the exoskeleton be under 2000 USD, not including those under 0.20 USD. Therefore only cheap materials and production techniques will be used to create the exoskeleton. Note that labor and tooling costs are not included, which would have a large impact in a more realistic setting.

#### 2.2.3 Safety

The Exo Games specifications stipulate it is required to keep a safety factor of at least 3 for critical components or to explain why a lower safety factor would be sufficient for certain components in the design. This rule is in place so no dangerous failure of the exoskeleton could occur during the competition. Since mostly 3D printed materials will be used special care will need to be taken to assure that the exoskeleton does not violate this limit. This was investigated in the final design using finite element analysis (FEA). Explosives or combustion can not be used in the exoskeleton design. If the design is active, it must include a kill mechanism (emergency stop button or something similar) to render the exoskeleton safe in case of an emergency. Finally, the user must be able to remove the exoskeleton within 1 minute without help from others.

## 3 Design

In order to determine the design requirements that will have to be respected the team looked at both task specific requirements and general design choices for ease of use and manufacturing.

### 3.1 Task-specific requirements

These are requirements that try to optimize the performance of the exosuit for the specific task that needs to be executed.

•For **warehouse palletization (ASTM F3443)** the test person needs to lift heavy objects. A back exosuit could assist this action, as well as improving the user’s posture (which is part of good task execution as mentioned before). Support would be given to the hips in order to take over some of the load from lifting. When the weights have to be carried over a certain distance arm support could be beneficial as well. Assistance would be provided to the arms by redirecting part of the load onto the shoulders.
•The **bomb squad walk (ASTM F3443)** could benefit from arm support. It will take some of the load from the arms when carrying the weights over an extended distance. This support should transfer the load from the arms to the shoulders, as in the palletization task. Leg support could also be beneficial to aid in this long walk attempting to take some of the load from the hips or the knee joints. These joints require more force to remain straight and as a result the walking is more tiring with the weights. For the bomb squad walk it is important that the user can carry the load close to his body. This will decrease the lever arm and allow the user to carry the weights for a longer time, as stated in the good execution description for this task. This means that there should be no protruding parts at the hips that hinder this carrying.
•For the **cup stacking** task there are no real physically demanding parts. The task requires mostly a lot of dexterity. This imposes that the exosuit should be easy to move around in and never be in the way or limit the movement of the user. Especially the arms of the user should not be hindered in any way. Protruding parts at the front of the exosuit could also prove to be a hindrance and result in poor task execution, so these should be avoided. Note that this is the only task that does not have an associated ASTM standard.
•The **timed up and go (ASTM F3528)** has one task that can be somewhat physically demanding for the knee, namely standing up from the chair. However, incorporating this into the exosuit would require an elastic element attached to the knee to store energy when sitting down, which would make this movement very uncomfortable and will only provide a limited advantage. As such, this type of support is not considered. When sitting down, it is also important that there are no bulky protruding parts of the exosuit that prevent the user from sitting down effectively or cause the chair to move, which could result in poor execution of the task. Freedom of movement is also very important in this regard.

Based on these tasks, it was decided that the exosuit should support the shoulder and lower back joint. These types of support can greatly relief the strain on the user’s lower back when the user is performing a lifting motion like stooping. Furthermore, the should support allows to remove the strain on the user’s arm when holding a load for a prolonged time.

### 3.2 Design choices

These requirements are not task specific or imposed by ASTM, but rather give directions for the overall cost, comfort and user friendliness of the design.

#### 3.2.1 Passive or active exoskeleton

A first choice that has to be made is whether an active or passive exoskeleton should be used. Since passive exosuits have a greatly decreased complexity and cost it stands out as the best choice to keep the price and prototyping time down.

#### 3.2.2 Support placement

The locations where support will be provided on the body by the exosuit have to be determined as well. The team wanted to focus on the palletization task in particular since it is one of the more demanding tasks on the body of the competition. Back support at the hip joint is useful for this and this kind of support will be integrated in the design. Arm support can also provide benefit for carrying the loads during palletization. As an additional benefit this support is also useful during the bomb squad walk.

#### 3.2.3 Material requirements

In order to iterate quickly during the design process, and because of the ease of access to materials, the team attempts to use as many 3D printed PLA parts as possible. These parts are cheap to make in small quantities and quick to prototype. Nuts, bolts and wheel casings will be made out of steel since these will be used in places where high stresses occur. The use of steel parts in these places will improve the safety of the exosuit and only cause a limited increase in price. For the springs a readily available option is needed and bungee cords provide a good solution for this.

#### 3.2.4 Comfort

Comfort is an important factor when designing an exosuit. In order to increase the comfort of the design the team proposed to make an exosuit that is as modular as possible. This way a user can modify the device when it is uncomfortable or when a task requires a different amount of support. An additional benefit of modularity will be that it can be adapted to different body shapes and types and as a result the exosuit can be more widely used.

#### 3.2.5 Minimal interference

The exosuit should not impede the user’s freedom of motion when the support it gives is not needed. For example, normal walking should not cause the back support system to be activated and movements close to the body should not be hindered by the arm support.

#### 3.2.6 Modularity and cost

Modularity and low cost are staples of the design and should be as present as possible. Some ways of achieving modularity will be proposed in the design and the cost should be kept as low as possible.

### 3.3 Physical design

**FIGURE 3:**
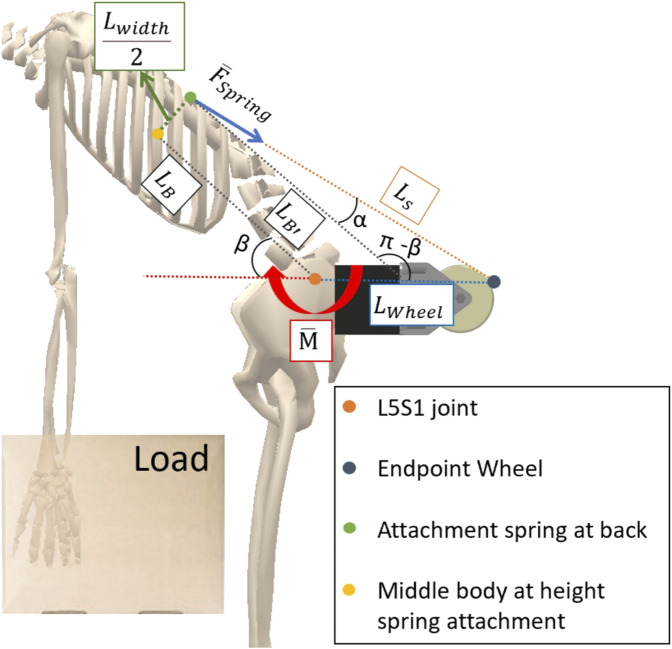
Free body diagram explaining how the back support of the exosuit generates a moment around the L5S1 spinal vertebrae.

The design was inspired by the HeroWear Apex exosuit that is commercially available. Just like the HeroWear Apex the exosuit uses back mounted elastic bands in order to provide back support. Potential gravitational energy is stored in the springs during a downwards motion and released when it is most needed during the upwards part of the motion against gravity. In order to better use the force delivered by the springs, wheels are mounted on the back of the exosuit to guide the force in a more horizontal direction. This means that a larger component of the force will produce a moment with the length of the upper back as a lever arm instead of only the thickness of the muscles and skin, thus creating a larger moment around the waist. A free body diagram is drawn to investigate if the exosuit could aid the user during lifting. This free body diagram can be found in Figure 3. Note that if the $L_{\mathrm{wheel}}$ distance would be increased the spring force will be more perpendicular to the surface of the back.

The moment $\vec{M}$ acts around the L5S1 vertebra of the spinal cord which is the lowest vertebra in the spinal cord. The moment caused by the spring force of the rubber bands $\vec{M}$ will be investigated. To investigate this moment we define some points on the free body diagram. The L5S1 joint, the endpoint of the wheel, the attachment point of the spring at the back of the body and the middle point of the body if we draw a line perpendicular to the back at the height of the spring attachment point. These points will help us to define some lengths in the free body diagram. $L_{B}$ gives the distance between the L5S1 disc and the middle of the body at the attachment point height. $L_{B^{\prime }}$ is the length parallel to $L_{B}$ starting from the attachment point of the spring. $\frac{L_{\mathrm{width}}}{2}$ gives the distance between the middle of the body and the attachment point of the spring. $L_{\mathrm{wheel}}$ gives the distance between the L5S1 vertebra and the wheel end point. For the calculations the assumption is made that $L_{B}$ , $\frac{L_{\mathrm{width}}}{2}$ and $L_{\mathrm{wheel}}$ have a constant length. We also assume that $L_{\mathrm{wheel}}$ will remain horizontal and that “Endpoint wheel” remains in the same place throughout the movement. The magnitude of the total moment around the waist generated by the device is given by:

$$M=F_{\mathrm{spring}}\left(\beta \right)\left(cos\left(\alpha \right)\frac{L_{\mathrm{width}}}{2}+sin\left(\alpha \right)L_{B}\right)$$

α in this equation is dependent on the bending angle of the user β as shown in Figure 3. The spring force is also dependent on β since the bending angle will determine the extension of the elastic bands, resulting in a variable force through Hooke’s law. A formula linking α and β needs to be found. This can be done by expressing the sum of angles the triangle formed between the attachment point of the spring, the endpoint of the wheel and $L_{B^{\prime }}$ . The angle between $L_{S}$ and $L_{\mathrm{wheel}}$ is still unknown and can be determined by using trigonometry and first looking for the lengths of the sides of this triangle $L_{S}$ , $L_{B^{\prime }}$ and $L_{\mathrm{wheel,2}}$ . $L_{\mathrm{wheel,2}}$ can be calculated by drawing a line parallel to $\frac{L_{\mathrm{width}}}{2}$ in the L5S1 vertebrae and using trigonometry in the resulting triangles:

$$L_{\mathrm{wheel,2}}=L_{\mathrm{wheel}}-\frac{L_{\mathrm{width}}}{2sin\left(\beta \right)}$$

The length $L_{B^{\prime }}$ can be calculated by using the same triangle as for $L_{\mathrm{wheel,2}}$ and by expressing it as the sum of $L_{B}$ and an additional length that can be calculated in this triangle:

$$L_{B^{\prime }}=L_{B}+\frac{L_{\mathrm{width}}cot\left(\beta \right)}{2}$$

The length $L_{S}$ can be calculated by connecting the attachment point of the spring and the L5S1 vertebrae. The two triangles that are then obtained can be used to derive a formula for $L_{S}$ . $L_{sv}$ and δ are intermediate variables used in these equations:

$$L_{sv}=\sqrt{\frac{L_{\mathrm{width}}^{2}}{4}+L_{B}^{2}}$$

$$\delta =Bgtan\left(\frac{L_{\mathrm{width}}}{2L_{B}}\right)$$

$$L_{S}^{2}=L_{\mathrm{Wheel}}^{2}+L_{sv}^{2}-2L_{\mathrm{Wheel}}L_{sv}cos\left(\pi -\beta -\delta \right)$$

A link between β and α can then be established by calculating the unknown angle in the triangle defined by $L_{S}$ , $L_{B^{\prime }}$ and $L_{\mathrm{wheel,2}}$ and expressing the sum of angles in a triangle:

$$\gamma =bgcos\left(\frac{L_{B^{\prime }}^{2}-L_{S}^{2}-L_{\mathrm{wheel,2}}^{2}}{-2L_{S}L_{\mathrm{wheel,2}}}\right)$$

α=β−γ

In the final design the $L_{\mathrm{wheel}}$ value was set to be 125 mm. $L_{B}$ of the exosuit pilot was measured to be 950 mm. $\frac{L_{\mathrm{width}}}{2}$ can be taken as 65 mm (Wehner et al., 2009). Using these values the support factor S of adding the protruding wheels can be calculated by comparing the moment without wheels, meaning $L_{\mathrm{wheel}}=\frac{L_{\mathrm{width}}}{2}$ , and the moment with wheels. This support factor is a function of bending angle β because of the link between α and β as expressed above. If no protruding parts are present the spring force will be along the back of the user. If the protruding part is present there will be a perpendicular component as well and α can be used to make the projection:

$$S=\frac{cos\left(\alpha \right)L_{\mathrm{muscular}}+sin\left(\alpha \right)L_{\mathrm{back}}}{L_{\mathrm{muscular}}}$$

**FIGURE 4:**
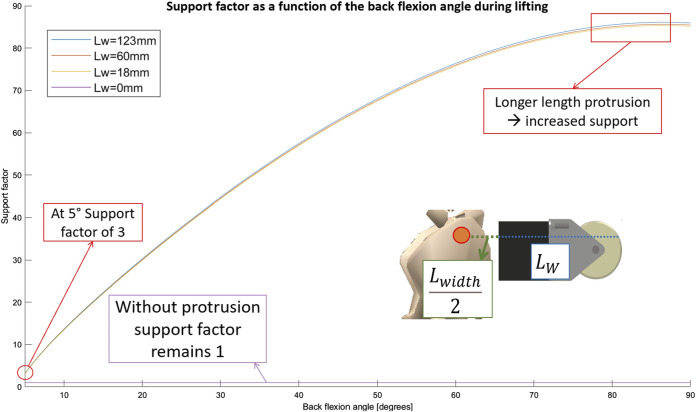
Support factor as a function of back flexion angle for different configurations of the $L_{\mathrm{wheel}}$ distance. Larger wheel distances increases the support provided. Note that the $L_{w}$ variable in the legend is equal to $L_{\mathrm{wheel}}-\frac{L_{\mathrm{width}}}{2}$ , not simply $L_{\mathrm{wheel}}$ , to simplify calculations.

A plot is made to show this support factor in function of the bending angle as seen in Figure 4. The benefit of using the wheels increases during the lifting motion up to values over 80 towards the end of the movement at 90° flexion. This explains the reasoning behind this design choice. As an additional benefit the spring does not chafe against the wearer’s body, resulting in a decrease in discomfort and an increased freedom of motion. This also means that a smaller spring force is needed for an equal amount of support. This is relevant since this spring force compresses the spine of the user which can be damaging in the long term if not minimized.

In order to provide greater flexibility the option was left to add 3D printed blocks at the bottom of the wheels, which can be used to elevate or lower them and change the $L_{\mathrm{wheel}}$ distance. This will increase the lever arm of the supporting moment and as a result decrease the compressive force on the spine. Figure 4 shows this effect of increasing or decreasing the $L_{\mathrm{wheel}}$ value. If it is decreased the support factor will be decreased, and *vice versa*. A similar effect of variable support could be achieved by increasing or decreasing the spring stiffness. This method would however increase the load on the spine when the support is increased. The separators allow the same effect without additional loading of the spine. The larger the $L_{\mathrm{wheel}}$ distance the more support is provided, thus also causing a larger force component to be perpendicular to the spine. Forces in this direction are much less detrimental to the back, but mitigating this effect can nonetheless be an interesting topic for future research.

**FIGURE 5:**
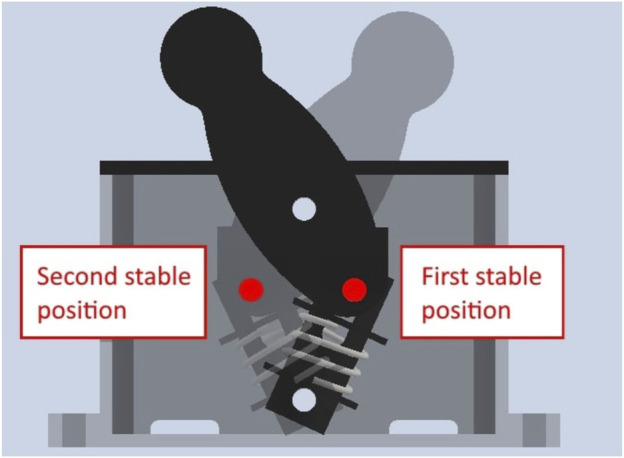
The bistable mechanism. The red dots show the two stable positions of the mechanism. When the exosuit is turned on or off, the mechanism switches from one stable position to the other.

The exosuit is equipped with an on/off switch that can be used to turn off the back support. This on/off switch aims to compensate for the low adjustability in the amount of provided support that is usually paired with passive exosuits. It gives the user the option to have no support when performing tasks that do not require any assistance. This mechanism works by using a system similar to the HeroWear Apex exosuit. Two springs are put in series: one stiff spring that normally provides support to the user and one compliant spring that is used to turn off the exosuit. When two springs are put in series the total stiffness of the mechanism is determined by the lowest spring stiffness. In our case this is the torsion spring that is only in in series when the exosuit is turned off. By either blocking or releasing this compliant spring the mechanism can be made stiff (on configuration) or compliant (off configuration). The torsion spring is blocked or released using a spring loaded bolt connected via a cable to a bistable mechanism that is used as a switch to actuate the bolt. Figure 5 shows the bistable mechanism in more detail. The switch moves a push/pull cable that can move the spring loaded bolt depending on which of the two stable positions the switch is in.

The stiff spring that is used when the device is on is a bungee cord, which are typically used to secure loads on a vehicle. These straps are cheap and come in many different lengths. The different available spring lengths give another chance for modularity. Carabiners are used as attachments between the springs and the exosuit, allowing them to be exchanged easily and quickly by the user. Different lengths of springs allow the user to modify the exosuit to the height of the user. This will result in a greater amount of comfort. When springs with different stiffnesses are used the amount of support provided can be modified. Together with the $L_{\mathrm{wheel}}$ distance, this now provides two separate ways to regulate support.

**FIGURE 6:**
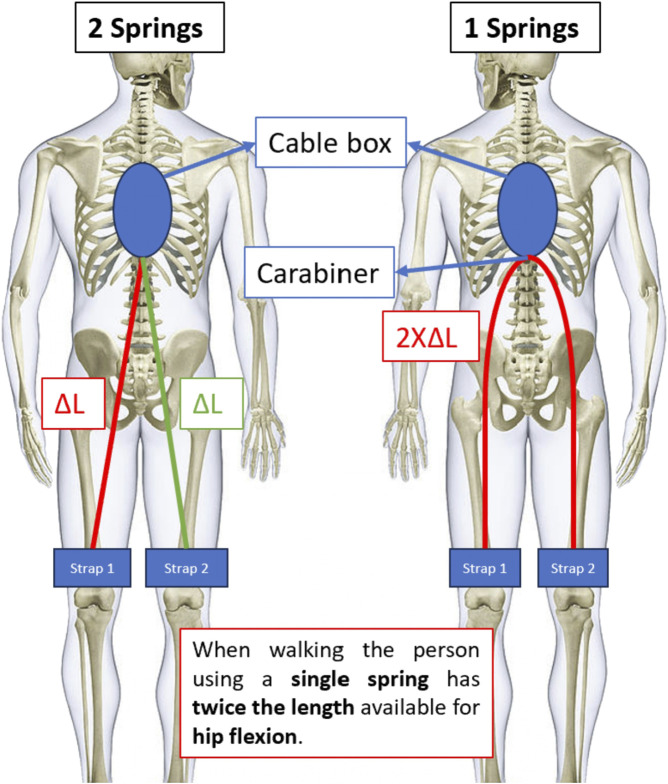
Comparison between using one or two bungee cords. During walking the person on the left has only Δ L distance to extend before feeling the spring resistance. The person on the right using a single spring has the Δ L on both sides available and can extend further before feeling resistance.

Since the bottom of the rubber bands is attached to the legs, issues could arise when walking if two separate springs are used. Each spring would extend when taking a step during normal walking and cause a force that works against the user’s intent. In order to somewhat mitigate this issue, and not interfere during any tasks that require walking, a single spring is used that attaches to both leg straps and is routed through a carabiner attached at the cable box. When either leg is pulled back, the distance between the spring attachment point and the carabiner shortens, while this distance increases when a leg is moved forward. In other words, during normal walking the changes in length of the single spring are compensated by one another. Figure 6 explains this in more detail. One of the problems with this lower back support is the mitigation of the straps. Due to the volume change of the leg throughout walking, the straps would come loose over time and started sliding downwards. This issue has been somewhat mitigated by buying better straps, but nevertheless, strap mitigation still occurred over time.

To provide arm support, springs are used running from attachments on the shoulders to easily releasable gloves that can be worn by the user. These springs are bungee cords, similar to the ones used in the lower back support. They are available in multiple lengths and stiffnesses, making this set-up adjustable for any wearer and any intended load that can be picked up. During the competition a bungee cord with a stiffness of 135.18 $\frac{N}{m}$ and a rest length of 50 cm. Thanks to this spring, when a user lifts a load the exosuit will help to hold the load up and transmit some of the force to the shoulders. Some padding is provided on the shoulders to increase the comfort for the user. Ropes are placed in parallel to the springs, which act as an end stop. These ropes are longer then the springs at rest. When too much extension of the springs occurs the full force of the load is transferred to the shoulders via these ropes and almost the entire force is taken from the user’s arms to the shoulders. This bypasses the arms completely and relieves them. The stiffness of the arm support is at first small but increases drastically when the ropes take over the load with a very high stiffness. The carrying of heavy loads is made easier with this parallel system.

A final modular part of the design are the leg straps. These straps are placed on the thigh of the user and the exact position can be adjusted until they are comfortable. The leg straps are an easy and secure way to attach the bottom parts of the rubber bands after they have been routed through the wheels.

### 3.4 Modularity

**FIGURE 7:**
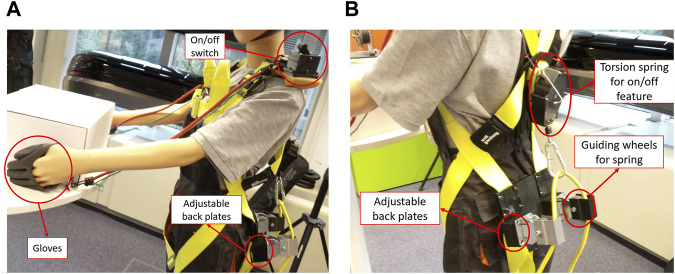
The front and back view of the finished exoskeleton design. **(A)** Front of the final design, with some features of the exosuit annotated. The adjustable back plates allow the user to change the Lwheel distance by adding or removing blocks. **(B)** Back of the final design with some of the features annotated.

In the end five different types of modularity are available to the user, which are briefly summarized here. Figure 7 shows this graphically.

#### 3.4.1 Adjusting safety harness straps

The first type of modularity is inherent to the base of the exosuit, the safety harness. Straps can be adjusted near the chest and between the legs to accommodate variance in body shape and size.

#### 3.4.2 Leg strap placement

The straps that attach the springs to the user’s legs can be moved up or down to make sure the springs are extended when bending over. If desired, they can even be used to pretension the springs.

#### 3.4.3 Spring length

The length of the springs for both the back support and arm support can easily be adjusted by replacing them by bungee cords of different sizes. Of course, the length can not be continuously varied in this way, but it should allow for enough adjustment to fit a large variance of people.

#### 3.4.4 Spring stiffnesses

Similar to how the length of the springs can be altered, their stiffness can also be changed by using a different bungee cord. Usually, the stiffness is inversely proportional to the length of the bungee cord, but one could also opt to use a different diameter.

#### 3.4.5 Adding/removing separators

The wheels guiding the springs are attached to the exosuit using bolts that pass through 3D printed separators. These separators can be swapped out for ones with a larger or smaller height, allowing to move the wheels closer to or further away from the user’s back (and change $L_{\mathrm{wheel}}$ as a result).

This modularity is important since different people have a different height, body proportions and personal preference. The support desired will depend on the person using the exosuit or on the task that has to be performed. The modularity of this exosuit allows a wide range of people to have a comfortable experience in a variety of tasks. Leg strap placement and the adjustment of the safety harness are fairly standard across different exosuits and passive exoskeletons. The other three are more specific to the design.

## 4 Lab experiments

### 4.1 Methods

The forces in the back springs and arm springs were measured in a variety of tasks in order to get an idea of the extent in which the exosuit will aid and hinder the user. Tests were performed using the ERGO FET from Hoggan Scientific that allows to measure forces up to 1335 N. This device can be used to measure both compression and tensile forces. The data can be streamed to a computer through Bluetooth. The force sensor is placed between the carabiner underneath the torsion spring and the back spring for all experiments except the palletization test. For the palletization test the force sensor is placed between the carabiner on the shoulder and the arm springs.

All experiments are performed on the same test subject, with the same $L_{\mathrm{wheel}}$ distance and choice of springs. This means that the force values achieved are comparable between experiments. The springs used during both the competition and these experiments have a stiffness of 135 $\frac{N}{m}$ . The rest length of the springs used is 50 cm. The subject is aged 24 years, with a height of 185 cm and weight of 65 kg. As a first experiment the test subject bends forward to 90° hip flexion. The test subject then moves back to the starting position with 0 degrees of hip flexion. The test subject repeats this three times in quick succession. To see if the exosuit would hinder the user when no assistance is required three tests are performed:

•Sideways flex test: the test subject will bend his torso sideways from side to side. He does this with increasing amplitude to determine the back spring forces generated.
•Torsional flex test: the test subject will twist his torso with increasing amplitude until he reaches the furthest possible twisting angle. The back spring forces are recorded.
•Sit to stand: For comfortable sitting, no forces are desired while the subject is sitting down. When moving up from or down to the chair, support could be beneficial, but our exosuit is not designed to support these movements and should thus not impart any forces throughout the experiment. This task is performed three times in succession and the back spring forces are again recorded.

**FIGURE 8:**
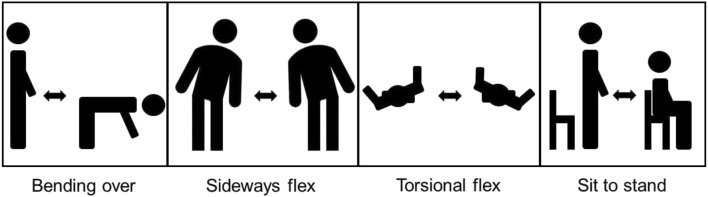
A graphical summary of each experiment. The palletization test procedure can be found in Figure 1.

These procedures are based on similar tests from (Govaerts et al., 2024). Finally, a palletization test is also performed. This is a simplified version of the one performed at the Exo Games due to limited resources. A box, weighing 7 kg, is lifted from the ground and carried for a distance of 5 m. A summary of the experimental tasks can be found in Figure 8.

### 4.2 Results

#### 4.2.1 Bending over

**FIGURE 9:**
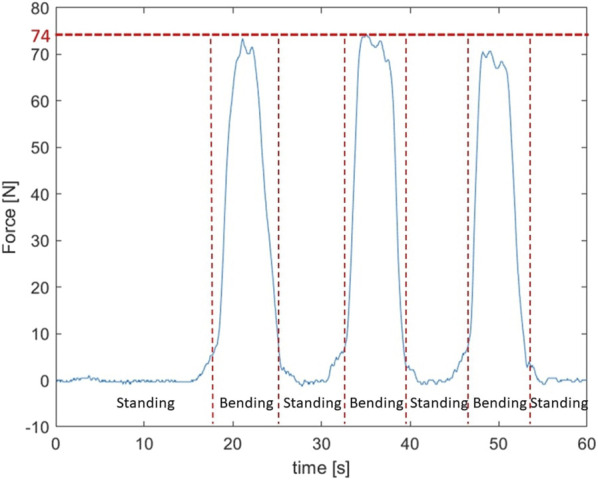
The test subject bends forward three times going to a maximal spring force of 74 N. This spring force is reached fairly consistently across the three movements.

From Figure 9 it is clear that the springs provide consistent assistance to the user when the same load is lifted since the shape and maximum of the curves are very similar. The maximal force that was measured is 74 N. If more support is required the $L_{\mathrm{wheel}}$ distance could be modified to use this force in a more efficient way by providing a larger lever arm as discussed before. Secondly the force itself could be increased by increasing the stiffness of the springs. For comparison, one study shows that the Laevo V2.4 passive back exosuit generates a peak force of around 85 N (Koopman et al., 2019), not counting the higher force required to bend the supporting structure of the exosuit itself when reaching the end of the range of motion. For this particular spring stiffness, force are thus generated in the same range as a commercial exosuit.

#### 4.2.2 Flex tests

**FIGURE 10:**
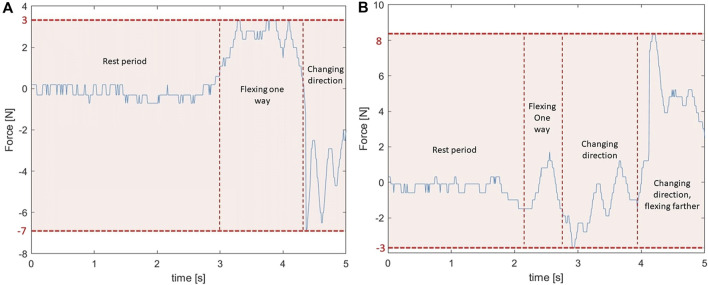
Back springs flex tests to see how much hindrance a user experiences. **(A)** The spring forces in the back springs created by moving sideways are minor and cause low hindrance. **(B)** The spring forces in the back springs created by moving torsionally.

The results of the two flex tests can be seen in Figure 10. All tests were performed with the exosuit turned off using the bistable mechanism.

For the sideways flex test the forces are limited between −7 N and 3 N. The minus values are strange at first sight since the rubber band springs used should not provide any compression forces. These negative forces are not caused by calibration errors since the sensor values always return to zero when no external force is applied. A possible explanation could be that the rather bulky sensor touched with another part of the exosuit providing friction and potentially compressive forces. Another explanation may be that the inertia of the sensor causes the force value to turn negative briefly when changing directions. This is possible seeing as this type of sensor is generally used for static force measurements, not dynamic ones. The full graph is provided but the negative values can be discarded for the analysis since they can not be caused by the bungee cords. The maximum of 3 N is very small and almost unnoticeable to the user. Compared to the maximal spring force during a lifting motion this is only 4.1%. This proves that the on/off feature can give an increased freedom of movement when required. High forces during this test would mean that a user feels a lot of resistance when moving.

The torsional flex test shows very similar results with a maximal force measured of about 8 N or 10.9% compared to the maximal force during lifting. The exosuit limits the freedom of motion a little more here, but only when the body is under maximal torsion towards the end of the experiment. This is because it is only then that the rubber bands are fully stretched out and can start applying forces.

#### 4.2.3 Sit to stand

**FIGURE 11:**
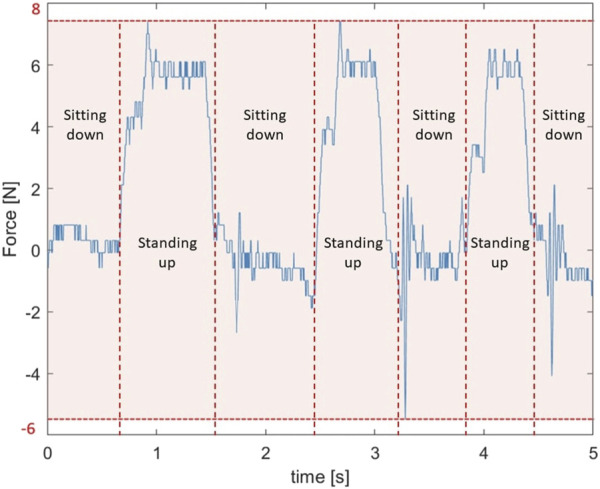
The spring forces in the back springs created by sitting up and down three times in quick succession.

The final test is sit to stand. This task should be performed at the Exo Games as well and is present in the competition to see how much the exosuit interferes with the user during an every day task like sitting down or standing up. As can be seen in Figure 11, the trials that were performed in succession show a very comparable behaviour. Maximal forces of around 6 N are measured or around 8.2% of the maximal lifting force. This does not prevent the user from sitting or standing up and even though the user feels this force constantly during sitting, as was indicated anecdotally by the test subject, it is not perceived as uncomfortable for them.

#### 4.2.4 Palletization

**FIGURE 12:**
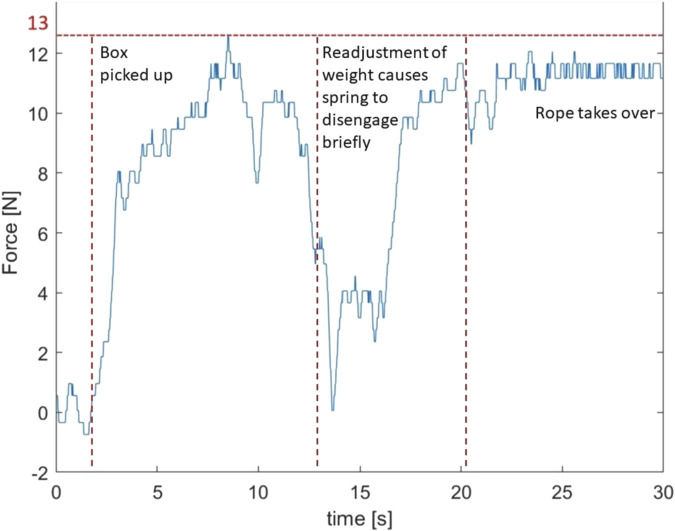
The spring forces in the rubber bands of the arms during a palletization task.

In Figure 12 the arm forces during the palletization test are shown. Peak forces of around 13 N are achieved. Due to the addition of the rope the maximal spring force generated during the task is limited and reaches a plateau during the carrying part of the task at the end of the graph. This is the effect of the end stop mechanism. The bungee cord supports the load until 13 N is reached, at which point the inelastic rope engages. This is also the maximal resistive force a user will feel when trying to perform a task without hand support where dexterity is most important. When the rope comes under tension, this higher stiffness takes over the remainder of the load. Spring forces were measured on only one side which means that the total force carried by the springs to the shoulders is around 26 N. Since the box weighed 7 kg the force carried by the rope to the shoulders will be 44 N.

## 5 Performance during the tasks

The exosuit was rigorously tested during the four tasks of the 2023 Exo Games. The findings about the performance of the exosuit are given here task by task, as well as a conclusion on the general performance across all tasks.

### 5.1 Task performance

#### 5.1.1 Timed up and go

The timed up and go was performed first. During the task it became clear that the protruding back wheels were not a big problem for sitting down and barely hindered the test subject or risked to disturb the chair. Since there was not much hindrance present during walking, the speed with which the task was completed relied more on the user itself. As per good task execution practice mentioned before, a moderate walking pace was maintained instead of trying to run as quickly as possible. The exosuit allowed to keep a good posture while sitting down and standing up, an important criteria when the task was evaluated by the judges.

#### 5.1.2 Bomb squad walk

From the start it was clear that the arm support provided great benefits. The ropes were engaged and made sure that the arms of the test person did not tire out when held to the side, according to good task execution practice. The gloves also had an effect since it helped to hold on to the handlebars of the weights and protected the hands from the weights digging in. Including gloves in exosuits for carrying tasks could be a good standard practice. When the pipes had to be turned around the back assistance was disabled and the greater freedom of movement allowed the task to be completed easily. Because the apparatus containing the pipes was placed next to a wall, it was not possible to move around it which is technically poor execution practice. Afterwards the device was turned on again and the second walk could continue.

#### 5.1.3 Cup stacking

Cup stacking proved not to be hindered by the exosuits when it was turned off. Turning it on may in theory be useful in relieving strain from the back caused by bending over for an extended period, but the wearer of the exosuit only bent over slightly (attempting to follow good execution practice) and did not find this uncomfortable. The cups used in the competition were paper cups instead of plastic competition cups. These paper cups are challenging to separate. The gloves themselves aided in the separating of the cups from the initial stack however, and the springs at rest were long enough not to hinder the user when performing the task. This aided us in setting the best time in the competition for cup stacking.

#### 5.1.4 Warehouse palletization

During the warehouse palletization task the exosuit could get benefits from the back support that is provided by the design. Unfortunately, poor task execution is promoted by the design of the exosuit, as bending over is a more natural way to extend the springs. Squatting provides similar levels of support however, though there is a chance of the bungee cords sliding along the legs to the sides of the user. The lifting of the boxes was made easier by the release of energy in the springs. This provides assistive force to the hip joint when trying to stand up again. The arm support then aided in carrying the boxes to the table and keep them at table height to allow an easy deposition of the box. A somewhat unexpected benefit of the gloves was that they could provide some additional grip as well as protecting the user against pinch points present on the boxes. This again shows the benefits of including gloves in exosuit and exosuits design, though this is true for any pair of gloves, regardless of the presence of the exosuits.

### 5.2 General findings

The exosuit proved to be a fairly comfortable design. The modular design allowed the two different users that performed the tasks to have a comfortable experience and to use different sets of rubber bands to get the desired amount of support. It was possible to put on the exosuit alone but in some cases help was provided to speed up the process. The limited number of protruding parts proved useful when trying to navigate through groups of people to get to the tasks. This is a realistic case since exosuit users in the real world will also need to be able to work next to people in close proximity without hurting or hindering them (Gonsalves et al., 2024).

The on/off switch of the exosuit proved to be unreliable and required frequent tweaking between the task. This was caused by too much slack on the cable that had to be adjusted frequently. This also meant that the on/off mechanism only really worked when the cable was under tension. This was only the case when the user was in a perfectly upright position. In future designs, this could be remedied by adding a system to pretension the cable properly, or by replacing the bistable mechanism with something that can pull the cable more than once. A possible candidate for this would be a ratcheting mechanism, similar to what is found on bike gear shifters.

## 6 Discussion

This section will describe some possible changes that can be made to the exosuit to further improve its performance. Some lessons learned during the competition will also be discussed.

•A first issue was the on/off system. Due to the bistable mechanism there are only two possible configurations, since there are only two stable positions. This means that if the distance change of the cable between these two stable positions is not perfectly calibrated to the user, the on/off feature will not work. The team from Texas A&M, whose exoskeleton only featured an elastic band based back support, used a different on/off mechanism in their design that featured a wheel on the shoulder that could be turned to roll or unroll a wire attached to the back springs. This system means that any wire length can always be rolled up enough for the system to turn off or back on again. This system is much better at accommodating different body sizes without the need for any adjustments.
•The team from the University of Central Lancashire (UCLan) had a similar system for the arm support but it featured an active motorized system to roll or unroll the rubber bands to a desired length automatically. This provides a continuous amount of lengths and requires no physical changes to the exoskeleton. This is in contrast to our design that only has a discrete amount of options for the length of the springs. The continuous design is obviously advantageous over ours to broaden the variance of body dimensions that can be adjusted for. The design was only active when adjusting the length of the springs but did not provide any active assistance to the user. Because of this, our exosuit had the advantage of being much cheaper and not relying on batteries or other types of external power. The springs were also attached to the wrists using bands around the arm, whereas in our design the springs were attached to the hands directly via gloves. Attaching to the wrists has the downside of not relieving them of the load carried. Globally speaking, the UCLan team’s design accomplished mostly the same functionality as ours at a higher cost and complexity. Still, some small quality improvements over our design were present in theirs, such as the continuous variability in spring length.
•Different teams provided support at different joints with their exoskeleton or exosuit designs. Table 1 summarizes this.

**TABLE 1:** Support types implemented by each team.

Type of support	Brazil	Clemson	Texas A&M	UCLan	VUB
Arm				✓	✓
Shoulder	✓				
Hip		✓			
Back		✓	✓	✓	✓

Evidently, back support was the most popular type, with some teams opting for a combination of multiple types. Our design was fundamentally most similar to that of the UCLan team. Most teams went for a passive design, only one team (Clemson) had a truly active hip joint support.

•The perceived usefulness of an exososuit or exoskeleton for the task that it is used for is very important for the user acceptance. If users do not feel like the device really helps they will not tend to use it for a long time (Elprama et al., 2022). Our test subject said during the palletization and bomb squad walk that the exosuit did provide a feeling of helping the user. During the tasks like cup stacking where an exosuit was not required it did not hinder the user in a significant way. This combination bodes well for user acceptance. Another important factor for user acceptance is comfort (Giustetto et al., 2021). This can also have an impact on the overall performance of the device (Govaerts et al., 2024). Our design was comfortable due to the high degree of modularity. This was evidenced by the fact that two different people performed the tasks during the competition. Only during the timed up and go the user had to sit closer to the edge of the chair which was perceived as slightly uncomfortable. Most other designs in the competition were equally comfortable due to the exosuit being built specifically for the test subject and by using soft materials for a lot of the components. The perception of safety is also very important. If a user thinks the device is unsafe to use he will not be inclined to use it (Gonsalves et al., 2024). All designs tested during the competition were perceived as safe due to the low power of the active components and the overall forces exerted by or on the exosuit being limited. Our design put an emphasis on safety, as was recommended by the competition guidelines (high factor of safety, low number of protruding parts, no pinch points, etc.).
•While the exosuit was not tested in an industrial setting, some conclusions can be drawn from the previous sections. During the palletization and bomb squad walk tasks, the assistance was appreciated. These tasks are most relevant for practical applications in industry, which seems to suggest its effectiveness in such settings. Palletizing and carrying weights over long distances are common actions in warehousing and manufacturing, for example. The experiments showed that minimal hindrance is observed when assistance is not required. One major downside, however, is the portrusion of the wheels. These may pose a potential snagging hazard. A possible solution is to allow the wheels to be folded inwards when not in use.

## 7 Conclusion

The design proposed by our team proved to be more than capable to deal with the challenges of the ASTM 2023 Exo Games. Most of our competitors chose passive designs and most designs had good user acceptance due to comfort, safety and usefulness. A strong point of our design was the freedom of movement and high modularity that provided a very comfortable experience for the user while still providing a good amount of support during palletization and bomb squad walk tasks. The measurements performed on the exosuit further prove this point by showing the forces generated during flexion which were very low and the forces in the back springs during lifting which were a lot higher. With some more work the modularity could be increased further to provide an even better user experience.

## Data Availability

The raw data supporting the conclusions of this article will be made available by the authors, without undue reservation.

## References

[B1] Abdelmomen M. Dengiz O. Hoda S. Tamre M. (2019). “Research on upper-body exoskeletons for performance augmentation of production workers,” in DAAAM proceedings (DAAAM international vienna). Editors Katalinic B. 1, 0904–0913. 10.2507/30th.daaam.proceedings.126

[B2] Abdoli E. M. Agnew M. J. Stevenson J. M. (2006). An on-body personal lift augmentation device (PLAD) reduces EMG amplitude of erector spinae during lifting tasks. Clin. Biomech. 21, 456–465. 10.1016/j.clinbiomech.2005.12.021 16494978

[B3] Aida T. Nozaki H. Kobayashi H. (2009). “Development of muscle suit and application to factory laborers,” in 2009 international conference on mechatronics and automation, Changchun, China, 09-12 August 2009 (IEEE), 1027–1032. 10.1109/ICMA.2009.5246279

[B4] ASTM (2024). Commitee Ait Exoskeletons and exosuits. Technical Committees.

[B5] Babič J. Laffranchi M. Tessari F. Verstraten T. Novak D. Šarabon N. (2021). Challenges and solutions for application and wider adoption of wearable robots. Wearable Technol. 2, e14. 10.1017/wtc.2021.13 38486636 PMC10936284

[B6] Crea S. Beckerle P. De Looze M. De Pauw K. Grazi L. Kermavnar T. (2021). Occupational exoskeletons: a roadmap toward large-scale adoption. methodology and challenges of bringing exoskeletons to workplaces. Wearable Technol. 2, e11. 10.1017/wtc.2021.11 38486625 PMC10936259

[B7] De Looze M. P. Bosch T. Krause F. Stadler K. S. O’Sullivan L. W. (2016). Exoskeletons for industrial application and their potential effects on physical work load. Ergonomics 59, 671–681. 10.1080/00140139.2015.1081988 26444053

[B8] De Vries A. Baltrusch S. De Looze M. (2022). Field study on the use and acceptance of an arm support exoskeleton in plastering. Ergonomics 66, 1622–1632. 10.1080/00140139.2022.2159067 36546707

[B9] Elprama S. A. Vanderborght B. Jacobs A. (2022). An industrial exoskeleton user acceptance framework based on a literature review of empirical studies. Appl. Ergon. 100, 103615. 10.1016/j.apergo.2021.103615 34847372

[B10] Giustetto A. Anjos F. V. D. Gallo F. Monferino R. Cerone G. L. Pardo M. D. (2021). Investigating the effect of a passive trunk exoskeleton on local discomfort, perceived effort and spatial distribution of back muscles activity. Ergonomics 64, 1379–1392. PMID: 33970812. 10.1080/00140139.2021.1928297 33970812

[B11] Gonsalves N. Akanmu A. Shojaei A. Agee P. (2024). Factors influencing the adoption of passive exoskeletons in the construction industry: industry perspectives. Int. J. Industrial Ergonomics 100, 103549. 10.1016/j.ergon.2024.103549

[B12] Goršič M. Song Y. Dai B. Novak D. (2021). Evaluation of the HeroWear apex back-assist exosuit during multiple brief tasks. J. Biomechanics 126, 110620. 10.1016/j.jbiomech.2021.110620 PMC8453127 34293602

[B13] Govaerts R. Bock S. D. Provyn S. Vanderborght B. Roelands B. Meeusen R. (2024). The impact of an active and passive industrial back exoskeleton on functional performance. Ergonomics 67, 597–618. PMID: 37480301. 10.1080/00140139.2023.2236817 37480301

[B14] Kim H. June Shin Y. Kim J. (2017). Design and locomotion control of a hydraulic lower extremity exoskeleton for mobility augmentation. Mechatronics 46, 32–45. 10.1016/j.mechatronics.2017.06.009

[B15] Koopman A. S. Kingma I. Faber G. S. De Looze M. P. Van Dieën J. H. (2019). Effects of a passive exoskeleton on the mechanical loading of the low back in static holding tasks. J. Biomechanics 83, 97–103. 10.1016/j.jbiomech.2018.11.033 30514627

[B16] Li-Baboud Y. S. Virts A. Yoon S. Shah M. (2019). “Towards standard exoskeleton test methods for load handling,” in 2019 wearable robotics association conference (WearRAcon), Scottsdale, USA, 25-27 March 2019 (IEEE), 21–27. 10.1109/WEARRACON.2019.8719403

[B17] Luo Z. Yu Y. (2013). “Wearable stooping-assist device in reducing risk of low back disorders during stooped work,” in 2013 IEEE international conference on mechatronics and automation, Kagawa, Japan, 04-07 August 2013 (IEEE), 230–236. 10.1109/ICMA.2013.6617923

[B18] Mohamed Refai M. I. Moya-Esteban A. Van Zijl L. Van Der Kooij H. Sartori M. (2024). Benchmarking commercially available soft and rigid passive back exoskeletons for an industrial workplace. Wearable Technol. 5, e6. 10.1017/wtc.2024.2 38510984 PMC10952052

[B19] Pesenti M. Antonietti A. Gandolla M. Pedrocchi A. (2021). Towards a functional performance validation standard for industrial low-back exoskeletons: state of the art review. Sensors 21, 808. 10.3390/s21030808 33530377 PMC7865790

[B20] Pinto-Fernandez D. Torricelli D. Sanchez-Villamanan M. D. C. Aller F. Mombaur K. Conti R. (2020). Performance evaluation of lower limb exoskeletons: a systematic review. IEEE Trans. Neural Syst. Rehabilitation Eng. 28, 1573–1583. 10.1109/TNSRE.2020.2989481 32634096

[B21] Toxiri S. Näf M. B. Lazzaroni M. Fernández J. Sposito M. Poliero T. (2019). Back-support exoskeletons for occupational use: an overview of technological advances and trends. IISE Trans. Occup. Ergonomics Hum. Factors 7, 237–249. 10.1080/24725838.2019.1626303

[B22] Upasani S. Franco R. Niewolny K. Srinivasan D. (2019). The potential for exoskeletons to improve health and safety in agriculture—perspectives from service providers. IISE Trans. Occup. Ergonomics Hum. Factors 7, 222–229. 10.1080/24725838.2019.1575930

[B23] Wehner M. Rempel D. Kazerooni H. (2009). Lower extremity exoskeleton reduces back forces in lifting. ASME 2009 Dyn. Syst. Control Conf. 2, 49–56. 10.1115/DSCC2009-2644

[B24] Zhu Z. Dutta A. Dai F. (2021). Exoskeletons for manual material handling – a review and implication for construction applications. Automation Constr. 122, 103493. 10.1016/j.autcon.2020.103493

